# PIWIL-2 and piRNAs are regularly expressed in epithelia of the skin and their expression is related to differentiation

**DOI:** 10.1007/s00403-020-02052-7

**Published:** 2020-03-12

**Authors:** Johannes Pammer, Heidi Rossiter, Martin Bilban, Leopold Eckhart, Maria Buchberger, Laura Monschein, Michael Mildner

**Affiliations:** 1 Clinical Institute of Pathology, Medical University of Vienna, Währinger Gürtel 18-20, 1090 Vienna, Austria; 2 Department of Dermatology, Medical University of Vienna, Vienna, Austria; 3 Department of Laboratory Medicine and Core Facility Genomics, Medical University of Vienna, Vienna, Austria

**Keywords:** PIWIL2, Hili, piRNA, ncRNA, Skin, Keratinocyte, Carcinoma

## Abstract

PIWI proteins play multiple roles in germline stem cell maintenance and self-renewal. PIWI-interacting RNAs (piRNAs) associate with PIWI proteins, form effector complexes and maintain genome integrity and function in the regulation of gene expression by epigenetic modifications. Both are involved in cancer development. In this study, we investigated the expression of PIWIL-2 and piRNAs in normal human skin and epithelial tumors and its regulation during keratinocyte (KC) differentiation. Immunohistochemistry showed that PIWIL-2 was regularly expressed in the epidermis and adnexal tissue with strongest expression in sebaceous glands. Cell culture studies revealed an association of PIWIL-2 expression with the state of differentiated KC. In contrast, the PIWIL-2 expression pattern did not correlate with stem cell compartments or malignancy. piRNAs were consistently detected in KC in vitro by next-generation sequencing and the expression levels of numerous piRNAs were regulated during KC differentiation. Epidermal piRNAs were predominantly derived from processed snoRNAs (C/D-box snoRNAs), tRNAs and protein coding genes. Our data indicate that components of the PIWIL-2—piRNA pathway are present in epithelial cells of the skin and are regulated in the context of KC differentiation, suggesting a role of somatic gene regulation. However, putative roles in the maintenance of stem cell compartments or the development of malignancy in the skin were not supported by this study.

**Electronic supplementary material:**

The online version of this article (10.1007/s00403-020-02052-7) contains supplementary material, which is available to authorized users.

## Introduction

The skin is the largest barrier against various physical, chemical or biological stresses, constituting the first line of defense of the body. This function is dependent on both epidermal and skin adnexal differentiation and maintenance [12]. Whereas the role of gene expression and regulation during epidermal development has been investigated in detail, the role of small non-coding RNAs other than microRNAs (miRNAs) in the homeostasis of the skin is largely unknown [3].

Small non-coding RNAs regulate gene expression by binding to AGO and PIWI proteins. AGO proteins have been shown to connect to miRNAs and small interfering RNAs (siRNA) and to regulate translation, mRNA degradation and heterochromatin formation. In contrast, PIWI proteins bind to piRNAs with a length of ~ 26–31 nucleotides [21].

The function of PIWI proteins and piRNAs in restricting the movement of mobile genetic elements in the germline is well known [21]. The mouse homolog to human PIWIL-2, Mili, has also been shown to bind to piRNAs [1] and has been implicated in transposon control and DNA methylation [25]. In addition, PIWI proteins play important roles in a variety of cellular processes including stem cell self-renewal, spermatogenesis, RNA silencing, translational regulation and chromatin remodeling [21]. In contrast, the significance of piRNA originating outside of transposable elements is to a large extent unknown [20, 40].

Small RNAs in the nucleus are involved in histone modification, gene expression and alternative splicing [22, 28], probably by binding to nascent RNA or genomic DNA [19, 28]. Recently, the expression of piRNAs has been demonstrated in different human somatic tissues [32], including the central nervous system and the haematopoietic system [26]. In lower level organisms, piRNAs are presumed to be involved in DNA methylation and histone modification [39]. Accordingly, piRNAs, whose role has been well established in germline maintenance, are now also regarded as post-transcriptional regulators of gene expression in somatic cells [20].

Furthermore, a growing number of studies have demonstrated that several piRNA and PIWI proteins are aberrantly expressed in neoplasias [14]. PIWI proteins are involved in the development of malignancies of squamous epithelia [11, 16] and may be regarded as biomarkers in several entities [15]. PIWIL-2 is essential for the proliferation and escape from apoptosis of a variety of cancer cells [27].

An essential role of miRNAs and long non-coding RNAs in skin wound healing [17] and differentiation [30, 51] has been revealed recently, but a possible role of piRNAs in the skin has not yet been shown. Since PIWIL-2 is known to play a role in the maintenance of germline stem cells as well as in the development of carcinomas [27, 53], we set out to look for a possible role of this protein and piRNAs in the skin. We investigated the expression pattern and possible association of PIWIL-2 with epithelial stem cell compartments and neoplasias. In addition, we demonstrated PIWIL-2 expression in KC cultures and investigated its regulation during KC differentiation in vitro. The possible binding partners, i.e. piRNAs, were profiled from normal KC using next-generation sequencing, and their expression was correlated to the differentiation state of KC. Gene targets for differentially expressed piRNAs were identified from a database of non-coding RNA (ncRNA) sequences [5].

## Methods

### Ethics statement

Human material was obtained in compliance with local laws and regulations and the responsible ethics committee has approved all experimental procedures (vote no. 1149/2011: isolation and culture of cells from and analysis of normal human skin biopsy samples; and 1498/2017: Expression und Regulation von PIWI-Proteinen und piRNAs in Epidermis und Hauttumoren). Written informed consent was obtained from the participants. Mice were sacrificed by cervical dislocation according to the animal welfare guidelines of the Medical University of Vienna, Austria. The study was approved by the Ethics Review Committee for Animal Experimentation of the Medical University of Vienna, Austria and the Federal Ministry of Science, Research and Economy, Austria (approval number: BMWF-66.009/0124-II/10b/2010).

### Immunohistochemistry

Surgical specimens were processed according to standard pathological procedures. Immunostaining for PIWIL-2 (moAb D-5, Santa Cuz biotechnology, Heidelberg, Germany) at a dilution of 1:40 was performed on the BenchMark ULTRA Automated IHC/ISH slide staining system (Ventana Medical Systems, Tucson, Arizona, USA) using a universal DAB detection kit after antigen retrieval with ULTRA Cell Conditioning CC1 (Ventana). The staining intensity was graded as negative (less than 3% of tumor cells reactive), weakly (< 10%), moderately (< 30%) or strongly (> 30%) positive. Staining intensity was assessed by two investigators independently.

### Cell culture

Human primary KCs from abdominal skin were prepared as described previously [13] and cultured under low-Ca^2+^ conditions (0.15 mmol/L) in serum-free KC growth medium-2 (KGM-2, Promocell) at 37 °C in 5% CO_2_. Cells were routinely passaged at a confluence of 60–80%. For differentiation, KC were cultured at 100% confluency for 7 days under high Ca^2+^ conditions (KGM-2 medium with 1.2 mmol/L Ca^2+^). HUVECs, HDMECs and A431 cell lines were cultured and stimulated as described previously [33, 34].

### Western blotting of primary human keratinocytes

Cells were lysed in 1% NP-40/PBS supplemented with 1 mM PMSF. After removal by centrifugation of the insoluble debris, the protein concentration of the supernatant was measured by the bicinchoninic acid (BCA) method (Pierce, Rockford, IL). Lysates were subjected to SDS-PAGE in 12% gels under reducing conditions. Subsequently, the proteins were electroblotted onto a nitrocellulose membrane (Schleicher& Schuell, Germany) at 0.8 mA/cm^2^ for 2 h. The membranes were dried and incubated in blocking buffer (5% non-fat dry milk in PBS) followed by immune overlay with anti-PIWIL-2 moAb (Santa Cruz, dilution 1:500) or GAPDH moAb (1:2000, Cell Signaling Technology, Cambridge, UK). After washing, bound moAb was detected with HRP-labeled sheep anti-mouse-IgG (Amersham Life Science, UK). The immunoreactions were visualized by chemiluminescence using SuperSignal™ West Dura Extended Duration Substrate (Thermo Fisher Scientific).

### Western blotting of murine tissue

Testes and preputial glands were prepared from mice immediately after sacrifice by cervical dislocation, KC were derived from the tail. The tissues were homogenized by sonication in a buffer that contained 50 mM Tris (pH 7.4), 2% SDS and complete protease inhibitor cocktail (Roche, Mannheim, Germany). Protein concentrations were measured as noted above. For Western blot analysis, 40 µg protein was loaded per lane on SDS polyacrylamide gels (ExcelGel SDS, gradient 8-18, Amersham Biosciences). The proteins were electrophoresed, blotted onto a nitrocellulose membrane using the Multiphor II Electrophoresis system (Amersham Biosciences) and stained with Ponceau dye. After removal of the dye by washing, the membranes were incubated at 4 °C overnight with mouse anti- PIWIL-2 (Santa Cruz, dilution 1:500). Subsequently, sheep anti-mouse IgG (NA931V, GE Healthcare Limited, UK) as secondary antibody conjugated to horseradish peroxidase at a dilution of 1:10,000 was applied. Enhanced chemiluminescence (ECL) reagent (ThermoFisher Scientific) was used to reveal the bands.

### Total RNA isolation and quantitative real-time PCR for small RNA-Seq

Total RNA was isolated from epidermis and keratinocytes using Trizol^®^ Reagent (Invitrogen, Carlsbad, CA) according to the manufacturer’s instructions. RNA was quantified using a NanoDrop-1000 spectrophotometer (Peglab, Erlangen, Germany) and RNA quality monitored by an Agilent 2100 Bioanalyzer (Agilent, Böblingen, Germany). RNA was reverse transcribed to cDNA (Biorad, Hercules, CA, USA) and PIWIL-2 expression was quantified by RT-PCR (Applied Biosystems 7500 Real-Time PCR System, Life Technologies, USA). Primers were designed using the Primer3 software. The PCR product was sequenced to prove specificity of the PCR.

### Small RNA-Seq

Quality control of RNA samples was performed using the RNA 6000 Nano Kit (for total RNA) and smallRNA Kit (for small RNA species) on a 2100 Bioanalyzer (Agilent). Sequencing libraries were prepared at the Core facility Genomics, Medical University of Vienna using the NEBNext Multiplex Small RNA Library Prep Kit for Illumina according to the manufacturer’s protocols (New England Biolabs). Libraries were QC checked for appropriate insert size on a Bioanalyzer 2100 using a High Sensitivity DNA Kit and quantitated using Qubit dsDNA HS Assay (Invitrogen). Pooled libraries had an average length of 150 bp and were sequenced on a NextSeq500 instrument (Illumina) in 1 × 75 bp sequencing mode. Reads with a read length > 5 bp were adaptor trimmed and trimmed from the 3′-end to a length of 50 bp using FASTQ Toolkit App from BaseSpace (illumina). The Small-RNA App from BaseSpace was used for read alignment against four reference databases (abundant, mature miRNA, other RNA, and genomic). The outputs contain hits to mature miRNAs, isomiRs, and piRNAs expressed as ‘counts’.

### Total RNA isolation and quantitative real-time PCR (qPCR) for KC differentiation experiments

Total RNA was isolated as described above and quantified using a NanoDrop-1000 spectrophotometer (Peglab, Erlangen, Germany). 200 ng RNA of each sample were reverse transcribed using iScript cDNA synthesis kit (Bio-Rad, Hercules, USA). Relative quantification was performed according to a previous publication [2] using the Light Cycler Master SYBR Green I kit (Roche Applied Science, Penzberg, Germany) on a Light Cycler 480 thermocycler. Primers were designed by Primer3 software (https://primer3.ut.ee/) and synthesized by Microsynth AG (Vienna, Austria). Samples were normalized to beta-2-microglobulin as an internal reference gene and analyzed according to the ΔΔCt method as described by Pfaffl [36]. The following primers were used: beta-2-microglobulin 5′-GATGAGTATGCCTGCCGTGTG-3′ and 5′-CAATCCAAATGCGGCATCT-3′; Filaggrin 5′-AAGGTTCACATTTATTGCCAAA-3′ and 5′-GGATTTGCCGAAATTCCTTT-3′; Loricrin 5′-GGAGTTGGAGGTGTTTTCCA-3′ and 5′-ACTGGGGTTGGGAGGTAGTT-3′; Keratin 1 5′-ATCAATCTCGGTTGGATTCG-3′ and 5′-TCCTGCTGCAAGTTGTCAAG-3′; Keratin 5 5′-CAAGCGTACCACTGCTGAGA-3′ and 5′-TCAGCGATGATGCTATCCAG-3′; Keratin 10 5′-GCTGACCTGGAGATGCAAAT-3′ and 5′-AGCATCTTTGCGGTTTTGTT-3′; Keratin 14 5′-GACCATTGAGGACCTGAGGA-3′ and 5′-GGCTCTCAATCTGCATCTCC-3′.

## Results

### PIWIL-2 is regularly expressed in normal human skin

**Fig. 1:**
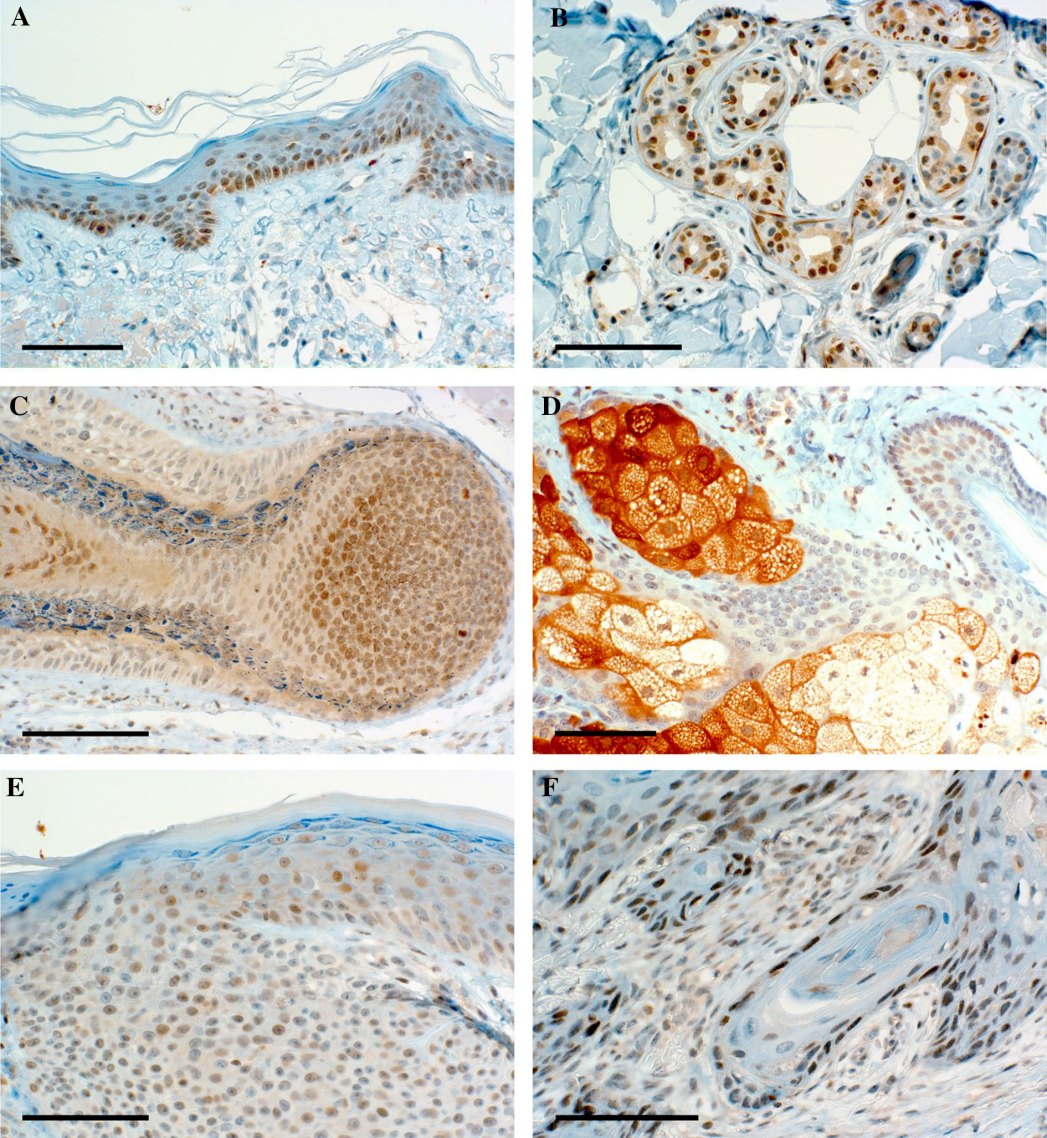
PIWIL-2 expression in human skin. An example of regular human skin showing moderate staining intensity for PIWIL-2 of keratinocyte nuclei (**a**). The inhomogeneous expression was mostly found in basal and suprabasal epidermal cells, the staining expression was usually weaker than in this example. Luminal cells of eccrine glands also stained weakly and inconsistently. In contrast, myoepithelial cells showed moderate cytoplasmic staining (**b**). Epithelial cells of the hair follicle were mostly negative, whereas this example shows weak to moderate reactivity for PIWIL-2 of the medulla (**c**). Strong and consistent expression of PIWIL-2 was found in mature sebocytes. In contrast, the basal layer of undifferentiated cells of sebaceous glands was negative to weakly positive (**d**). A basal cell carcinoma shows a staining intensity comparable to that of regular epidermal cells (**e**). Peripherally located undifferentiated cells of a squamous cell carcinoma stain moderately for PIWIL-2 (**f**). Scale bar = 100 µm

Nuclei of KC in healthy human skin were weakly to moderately positive for PIWIL-2. The expression was mostly found in basal and suprabasal cells of the epidermis and was inhomogeneous (Fig. 1a), whereas the upper layer was mostly negative. The expression was negative in 3 of 26 samples, weak in 15 and moderate in another 8 samples. A weak and inconsistent staining was also found in the minority of nuclei of luminal cells of eccrine glands. In contrast, myoepithelial cells showed moderate cytoplasmic staining (Fig. 1b). Epithelial cells of the medulla of the hair were inconsistently weakly to moderately reactive for PIWIL-2, whereas most other follicular epithelial cells were negative or rarely weakly reactive (Fig. 1c). The only cells of the skin with strong and consistent expression of PIWIL-2 were mature sebocytes. In contrast, the basal layer of undifferentiated cells of sebaceous glands was negative to weakly positive (Fig. 1d). Immunostaining was verified by RT-PCR showing specific PIWIL-2 mRNA expression in KC and the epidermis (data not shown).

Smooth muscle cells of both M. arrectores pilorum and the Tunica media of blood vessel showed moderate cytoplasmic staining (not shown).

### PIWIL-2 is not upregulated in basal cell carcinomas and squamous carcinomas of the skin

PIWIL-2 overexpression has been reported to play a critical role in the development of a variety of carcinomas [14]. We investigated the expression of PIWIL-2 in 9 basal cell carcinomas. Five were negative, whereas four showed weak expression (Fig. 1e). There was no upregulation in comparison to non-neoplastic epidermis of the same specimen.

Similarly, there was no upregulation of PIWIL-2 in squamous cell carcinomas. In 9 of 16 samples, the expression was weaker and in 5 samples, the expression intensity was similar in comparison to non-neoplastic epidermis. Only two carcinomas showed a slight upregulation of PIWIL-2 (Fig. 1f). The expression was most prominent in peripherally located undifferentiated cells, but was also found in some mitotic figures and in parakeratotic nuclei.

### PIWIL-2 is expressed in murine and human epidermal KC and sebaceous glands

**Fig. 2:**
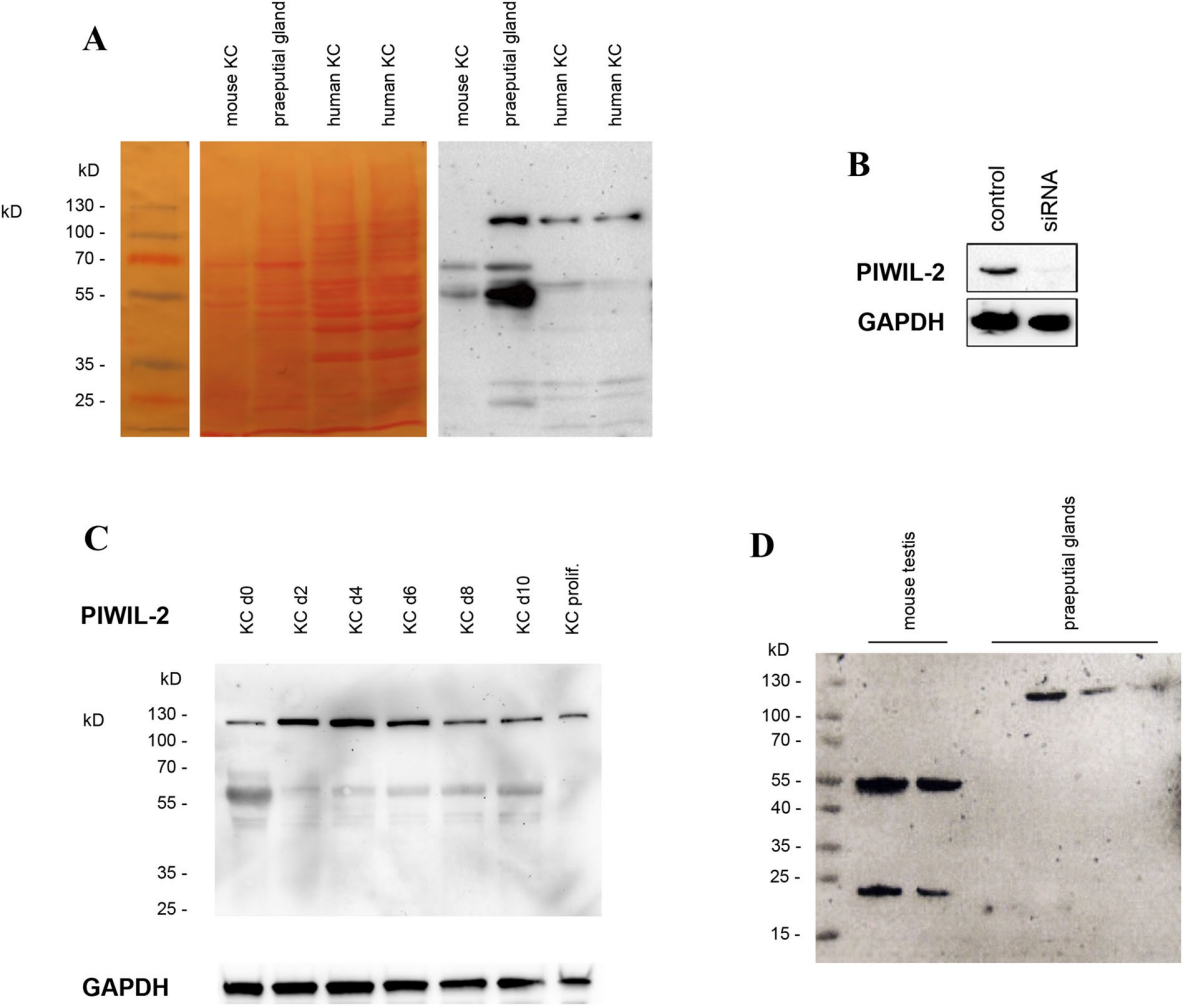
PIWIL-2 expression in murine and human keratinocytes and sebaceous glands by Western blotting. **a** Mouse preputial glands showed 130 kD PIWIL-2 bands, corresponding to the full-length protein as well as additional bands of ~ 55–60 and 20 kD corresponding to splice variants. Mouse KCs contained only smaller PIWIL-2 protein species. Human KC showed bands ~ 130 kD, corresponding to the full-length protein and only weak staining for smaller bands. **b** Treatment of human KCs with a specific siRNA against PIWIL-2 led to the disappearance of the PIWIL-2 band on Western blots. **c** In KC induced to differentiate in confluent culture PIWIL-2 expression increased during the first 4 days and later decreased. **d** In contrast to mouse preputial glands, mouse testis contained only PIWIL-2 protein species of 60 and 20

Next, we investigated the expression of PIWIL-2 protein in murine preputial glands, i.e. the largest type of sebaceous glands of the mouse, and primary human and murine KC. In preputial glands, PIWIL-2 bands of 130 kD, corresponding to the full-length protein, and bands of 55 and 20 kD were detected (Fig. 2a). Mouse KC (Fig. 2a) and testis (Fig. 2d) contained only PIWIL-2 protein species of ~ 55–60 and 20 kD, whereas human KC showed bands with the size of ~ 130 kD corresponding to the full-length protein and only weak bands of smaller molecular weights, mainly at 60 kD. PIWIL-2 proteins of approximately 60 kD and ~ 20 kD (Fig. 2a) were reported previously to correspond to splice variants of PIWIL-2 [50]. Accordingly, primary KC in cell culture expressed PIWIL-2 consistently. In addition, PIWIL-2 was expressed in human endothelial cells [HUVECs (3/3) and HDMECs (1/1)], but not in the autonomously growing squamous cell carcinoma cell line A431 (0/2) (data not shown).

Treatment of human KC with a specific siRNA against PIWIL-2 led to the disappearance of the PIWIL-2 band on Western blots, validating the antibody against PIWIL-2 protein (Fig. 2b). siRNA-mediated depletion of PIWIL-2 did not impair growth and survival of the cells in vitro (data not shown).

When KC were induced to differentiate by maintaining them in confluent culture for up to 10 days [7], the expression of PIWIL-2 increased during the first 4 days and later decreased again (Fig. 2c). This pattern of regulation was found in three independent experiments, suggesting a consistent induction of PIWIL-2 expression during early KC differentiation and a repression at the late stages of KC differentiation. However, preliminary experiments showed that PIWIL-2 knockdown does not lead to a significant regulation of the KC differentiation markers keratin 1, 5, 10 and 14 as well as loricrin and filaggrin (supplementary information, Fig. 1) in monolayer cultures.

### Screening of piRNAs expressed in human KC and regulated during differentiation

**Fig. 3:**
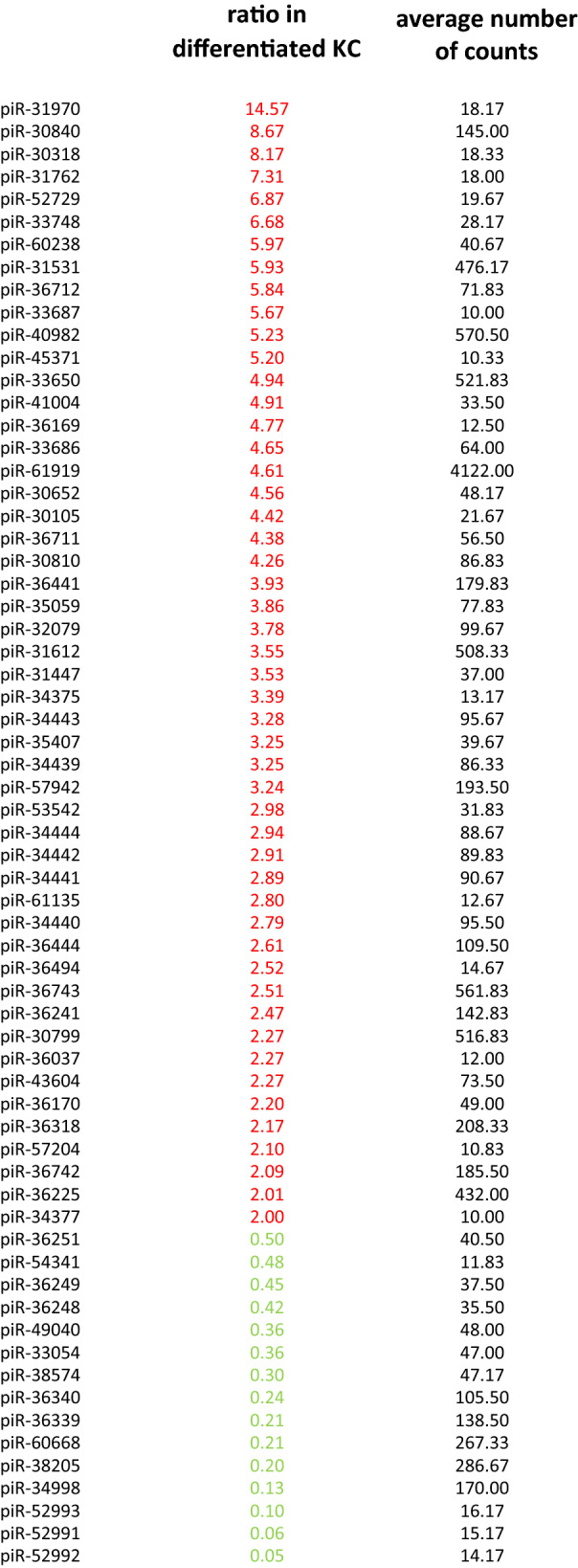
piRNAs expression is associated with the state of differentiated KC. Whereas 50 piRNAs were upregulated at least twofold (mean) in differentiating KC, 15 piRNAs showed a downregulation in differentiating KC by less than half

When investigating the expression of 3848 different piRNAs in human primary KC, we found 190 to be constitutively expressed using an arbitrary threshold of 10 counts in 6 samples consisting of undifferentiated and differentiated KC from 3 donors (Supplementary information, Table 1). 50 piRNAs were upregulated at least twofold (mean) in differentiating KC in three independent experiments (shown in Fig. 3). 29 of these 50 piRNAs showed a more than twofold upregulation in samples of all three donors and the average ratio was 4.19. In contrast, only 15 piRNAs showed a downregulation in differentiating KC by less than half, 9 of those in samples from all the donors (Supplementary information, Table 2).

piRNAs can be derived from other RNAs including tRNAs [9, 23] and snoRNAs [54] and may regulate their function by homologue interaction. When comparing the sequence of 100 piRNAs with the strongest expression level to non-coding RNAs, we found 39 of them to be homologous to tRNAs, 27 to snoRNAs (SNORDs, mostly C/D-box snoRNAs) and 5 to miRNAs (Supplementary information, Table 3). Three of these five piRNAs were homologous to let7 and these were some of the piRNAs with the highest expression level.

Whereas only 4 of 39 piRNAs homologous to tRNAs showed homology to protein coding mRNAs, 18 piRNAs homologous to SNORDs showed homology to protein coding mRNAs (Supplementary information, Table 2).

Upregulated piRNAs were more likely to be homologous to SNORDs (29/50) than to tRNAs (13/50) and 21 of them were homologous to 16 different proteins. In contrast, downregulated piRNAs were rarely homologous to SNORDs (1/15), or to tRNAs (3/15) and 3 of them were homologous to protein coding mRNAs.

piRNAs amplified by the ping–pong cycle are involved in transposon suppression and have been reported to have a uracil residue (U) at position 1 at their 5′-termini and an adenine residue (A) at position 10 [44]. Of 100 piRNAs with strongest expression in KC, 6 have been found to have this signature, whereas 29 had a U at their first position (Supplementary information, Table 3). 11of 50 piRNAs upregulated in differentiating KC had a U at position 1, 3 of these in combination with an adenine at position 10. 8 of the downregulated piRNAs had a U at position 1 and 4 of these in combination with an adenine at position 10. This distribution indicates a random frequency of these nucleotides.

Finally, PIWIL-2 knockdown resulted in the regulation of piRNAs in preliminary experiments (supplementary information, Fig. 1).

## Discussion

In this study, we describe that PIWIL-2 is regularly expressed in epidermal KC, epithelial cells of skin appendages as well as in mesenchymal cells. Our results are complementary to a variety of previous studies that do not show any PIWIL-2 expression in adult tissues [25, 45].

PIWIL-2 has been shown to be expressed in human germ cells and to play a role in maintaining genomic integrity by suppression of transposon activity [38]. In contrast, a possible function of PIWIL-2 protein and piRNA expression in KC and skin appendages has not yet been investigated. Similar to their activity in germ cells, PIWIL-2 and piRNAs could act on transposons and retroviruses in the skin. However, the very small number of piRNAs expressed in KC in vitro argues against a role in post-transcriptional control of a large variety of transposable elements (TE). Our results match those of a previous study showing that the population of piRNAs present in Hela cells is dramatically small in contrast to the enormous population of known piRNAs in male germ cells [31].

PIWIL-2 expression has been shown to be associated with stem cell properties and has been regarded as a potential neoplasia biomarker [41, 42, 53]. Intriguingly, we could demonstrate that in adult skin, PIWIL-2 is not predominantly expressed in compartments with possible stem cell populations in the epidermis. In fact, we even detected PIWIL-2 expression in differentiated sebocytes. Since c-MYC can be induced by PIWIL-2 in epithelial cells [49] and its overexpression stimulates differentiation in sebaceous glands [47], the observed strong PIWIL-2 expression may indicate that this protein is involved in sebocyte differentiation.

PIWI proteins are associated with chromatin remodeling by histone acetylation and methylation [11, 25, 27, 46, 52], and PIWI deficiency has been shown to drastically change the epigenetic landscape in Drosophila [18]. Accordingly, PIWIL-2 could be involved in KC proliferation and differentiation dependent somatic gene regulation. Strikingly, we did not observe altered expression of important KC differentiation markers. However, since KC in monolayer cultures do not fully differentiate, other, more sophisticated models, such as in vitro skin models or knock-out animals, would be necessary to address this question.

Recently, a role of PIWIL-2 in the repair of UV-induced DNA damage was identified by showing that UV irradiated mouse fibroblasts lacking PIWIL-2 protein expression were more susceptible to apoptosis [52]. However, we were not able to show a regulation of PIWIL-2 neither by UV-A nor by UV-B radiation in human KC (not shown).

Whereas PIWIL-2 upregulation has been described in precancerous stem cells [29] and in several malignant neoplasms [27, 37] suggesting a role in tumor initiation, we did not find a distinct upregulation of this protein in epithelial skin tumors. In contrast, we found a variety of piRNAs to be expressed in KC from healthy skin. Of 190 piRNAs identified in KC in this study, 33 have previously been described to be expressed in squamous epithelial cells [24] supporting the specificity of our findings. Presently, the significance of piRNA originating outside of transposable elements is largely unknown and piRNAs have already been shown to play a crucial role in host gene regulation [38]. In line with these publications our results suggest that piRNAs are also involved in epigenetic processes in adult epithelia [35].

Apart from homology to a variety of protein coding mRNAs, piRNAs identified in this study were homologous to miRNAs, i.e. let7 and miR-182, SNORDs and tRNAs. A major function of let7 genes may be to promote terminal differentiation [8, 10] and a recent study has identified let7 as a key mediator linking stemness genes with Lin28 in squamous cells also [4]. In addition, miR-182 has multiple roles in differentiation, development, and functional maintenance [6, 48]. The strong expression of let7 and miR-182 homologous piRNAs in comparison to other piRNAs indicates a role of these ncRNAs in the regulation of stemness and differentiation in KC, however, their exact function has still to be determined. Small nucleolar RNAs (snoRNAs) are known to target 2′-*O*-methylation of rRNAs. Since SNORDs [9] have been shown to be precursors of piRNAs [54], they may function as gene expression modulators [9, 43]. We also found the expression of abundant tRNA-derived small RNA (tsRNAs) in primary KC. Since some tsRNAs match known piRNA sequences and can be bound by the PIWI proteins, tsRNAs might also be involved in gene regulation [23].

In summary, we find PIWIL-2 and piRNAs to be constitutively expressed in the skin and KC in vitro. Our finding that PIWIL-2 expression is associated with the degree of differentiation in KC cultures and that a multitude of piRNAs is distinctly regulated in the context of KC differentiation suggests a role of somatic gene regulation by PIWI-piRNA pathways both in the epidermis and appendages of the skin. In contrast, no upregulation of PIWIL-2 in carcinomas was found. Our analysis of piRNAs expressed in non-differentiated and differentiated KC builds a basis for further studies investigating the role of these piRNAs in KC differentiation and epithelial homeostasis.

## Electronic supplementary material

Below is the link to the electronic supplementary material.

Supplementary file1 (XLSX 19 kb)

Supplementary file2 (XLSX 17 kb)

Supplementary file3 (XLSX 20 kb)

Supplementary file4 (psd 319 kb)
